# Inorganic Solid Electrolyte Interphase Engineering Rationales Inspired by Hexafluorophosphate Decomposition Mechanisms

**DOI:** 10.1021/acs.jpcc.2c07838

**Published:** 2023-01-23

**Authors:** Dacheng Kuai, Perla B. Balbuena

**Affiliations:** † Department of Chemical Engineering, Texas A&M University, College Station, Texas 77843, United States; ‡ Department of Chemistry, Texas A&M University, College Station, Texas 77843, United States; § Department of Materials Science and Engineering, Texas A&M University, College Station, Texas 77843, United States

## Abstract

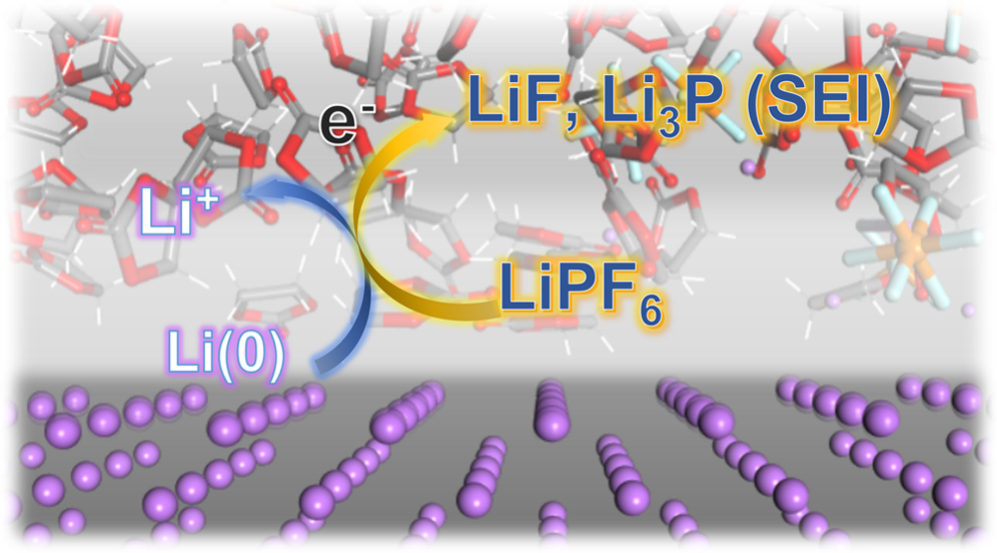

Solid electrolyte interphase (SEI) engineering is an efficient approach to enhancing the cycling performance of lithium metal batteries. Lithium hexafluorophosphate (LiPF_6_) is a popular electrolyte salt. Mechanistic insights into its degradation pathways near the lithium metal anode are critical in modifying the battery electrolyte and SEI. In this work, we elucidate plausible reaction pathways in multiple representative electrolyte systems. Through ab initio molecular dynamics simulations, lithiation and electron transfer are identified as the triggering factors for LiPF_6_ degradation. Meanwhile, we find that lithium morphology and charge distribution substantially impact the interfacial dissociation pathways. Thermodynamic evaluation of the solvation effects shows that higher electrolyte dielectric constant and lithiation extent profoundly assist the LiPF_6_ decomposition. These findings offer quantitative thermodynamic and electronic structure information, which promotes rational SEI engineering and electrolyte tuning for lithium metal anode performance enhancement.

## Introduction

Current commercially available lithium-ion batteries are approaching their theoretical limits, while the electronics market still urges further energy capacity extension.^1^ The lithium metal battery (LMB) is a promising technical route for next-generation energy storage devices.^2^ The cycling efficiency and safety risk control on the battery anode are the primary technical obstacles that hinder LMB commercialization.^3^ Solid electrolyte interphase (SEI) formed on the anode surface regulates Li^+^ ion migration and deposition, which is critical in minimizing lithium dendrite and whisker formation.^4^ Therefore, the battery’s electrochemical performance is tightly bonded with SEI morphology and composition.^5^

Fluorination has been suggested as an efficient strategy in battery electrolytes and SEI engineering.^6^ The fluorine-containing electrolyte components, such as LiPF_6_, lithium bis(fluorosulfonyl)imide (LiFSI), and fluoroethylene carbonate (FEC), experience decomposition and yield lithium fluoride nanoparticles in SEI. However, the SEI internal structures are notoriously complex to resolve due to their fragility and chemical reactiveness.^2^ So far, only limited while expensive techniques such as cryo-electron microscopy are able to precisely probe the spatial distributions of SEI nanostructures, where battery disassembly is mandatory.^7^ In addition, the electrolyte species also alter the solvation environment, which profoundly impacts the thermochemistry and kinetics of SEI formation reactions.^8,9^ Aside from the designing principles based on empirical concepts, theoretical approaches are expected to envision the electrolyte–electrode interfacial chemistry and ion transport mechanisms.^3,10−12^ Hereby, we study the reactions resulting in prevalent inorganic components in SEI from LiPF_6_-based electrolytes. The quantitative understanding of the reaction thermodynamics may enable future multiscale simulations of the SEI formation processes.

It is inherently challenging to characterize the electrode–electrolyte interfacial chemistry at the nanoscale.^13^ The SEI grows in such a highly reductive and salty environment. Despite limited experimental data available to precisely elucidate the SEI composition and formation mechanisms, many proposals depict plausible scenarios in generating SEI of LMB.^2,13^ Hexafluorophosphate degradation is recognized as the primary contributor to the LiF in SEI based on this electrolyte chemistry, while few reports denote the destination of phosphorus. A trace amount of moisture in battery electrolyte can cause hydrolysis of PF_6_^–^, yielding POF_3_ and its derivatives as detected in spectroscopic analysis.^14,15^ Further information regarding the direct PF_6_^–^ decomposition and reduction without water participation is less reported in the experimental work. The characterization of interfacial chemistry is particularly challenging in accessing delicate nanoscale surfaces.^13,16^ First principles simulation is compelling in predicting the molecular behavior at the metal–liquid interface, which is fragile to atmospheric interruptions.^17^ Ab initio molecular dynamics (AIMD) successfully predicts the stabilities and reductive dissociation of electrolyte solvents near metallic surfaces.^18,19^

The LiPF_6_ degradation chemistry near the anode shows unique features. The phosphorous element usually demonstrates a singlet electronic state in stable compounds. However, some reported atomic charge analysis on the AIMD simulation shows that this rule may not apply to all cases in the LiPF_6_ decomposition near lithium battery anode surfaces. Martinez de la Hoz et al. identified the plausible oxidation state pathway on the silicone anode surface, where the equilibrium charge state of phosphorous was identified to be −1.^20^ A fully reduced phosphorous reduction product was found in the lithium metal case.^21^ Both these scenarios require continuous electron uptake.

Ether-based electrolytes are mainly composed of dimethoxyethane (DME) and 1,3-dioxolane (DOL), with moderate dielectric constants (ε) varying from 4 to 10.^22^ High salt concentration is mandatory in maintaining the integrity of ether solvents in high voltages, despite their compatibility with lithium metal.^23,24^ The commonly used carbonate solvents, including ethylene carbonate (EC), propylene carbonate (PC), and vinylene carbonate (VC), create a solvation environment with much higher dielectric constants and unique solvation structures.^22^ In the electrolyte environment with a positive dielectric constant, the direct electron tunneling probability decays exponentially with respect to particle–metal-surface distance. Consequently, electron-induced reactions usually occur via complex charge transport mechanisms that manipulate the corresponding redox reaction rate.^25^ The ion–solvent interactions are one of the most important factors in bottom-up LMB development.^26^ The beginning stages of phase separation and ionic nucleation in forming SEI have been reported in previous molecular dynamics (MD) and AIMD studies.^27−29^ A longer simulation time scale may compensate for this issue by implementing high-quality ab initio thermodynamic information in kinetic Monte Carlo (kMC) simulations.^30^ Beyond elucidating the underlying chemical processes, we aim to contribute toward data-driven LMB electrolyte and SEI engineering from the electrolyte solvation and anode morphology perspectives.

## Computational Methods

After creating a vacuum slab on top of the Li metal crystal surface structure, the initial configurations for AIMD were obtained by packing the geometry-optimized structures of electrolyte species in the vacuum space based on the Amorphous Cell Packing module in Materials Studio v8. In the selected configuration, relative positions of hexafluorophosphate particles were verified to include both the following conditions: (1) close contact to the Li metal surface and (2) dissolved in electrolyte while the mass center is at least 5 Å away from the lithium surface. The molar ratios of each species and the system density were further assessed to be consistent with the experimental macroscopic properties. The AIMD simulations were performed via VASP 5.4.4.^31^ The Perdew–Burke–Ernzerhof (PBE) functional was used to describe the electron exchange and correlation energies within the generalized gradient approximation (GGA).^32^ Electron–ion interactions were considered within the projector augmented wave (PAW) pseudopotentials.^33,34^ The plane waves were extended to a cutoff energy of 520 eV. The simulations were based on an NVT ensemble at 300 K with 1-fs time step. DFT-D3 method of Grimme with the zero-damping function was implemented to correct van der Waals interaction terms.^35^ The atomic Bader charges were analyzed based on the grid-based method developed by Henkelman et al.^36^

Molecular modeling was primarily conducted on the Gaussian16 package. All intermediate structures were optimized to their local minimum without imaginary vibration mode, and the corresponding free energy values were converted to the standard state of 1 M. M06-2X/aug-cc-pVDZ level of theory was employed based on the benchmark results previously reported by Han et al.^37−39^ Solvation effects were evaluated based on the implicit solvation model based on density (SMD).^40^ Empirical dispersion interactions were introduced in calculations.^41^

## Results and Discussion

We initially estimate the degradation energetics of LiPF_6_ with different spin states at the molecular level, as shown in Table S1 in SI. The only free energy barrier exists at the first P–F cleavage process. However, the real SEI formation environment is much more complex than this simple model. As an example of the complexity of interfacial reactions, it has been reported that the solvent–reactant interactions at the air–liquid interface can profoundly alter the reaction thermodynamic profile, and such impact varies under different dielectric environments.^16^ Similarly, many factors, including metal–electrolyte interfacial effects, solvation effects, charge transport, and lithium coordination, contribute to the LiPF_6_ degradation reactions. Here, we apply AIMD simulations to elucidate the interfacial decomposition reactions and further quantitatively evaluate the key conditions at the molecular level.

### Lithiation and Electron-Tunneling-Induced Salt Degradation

**Figure 1:**
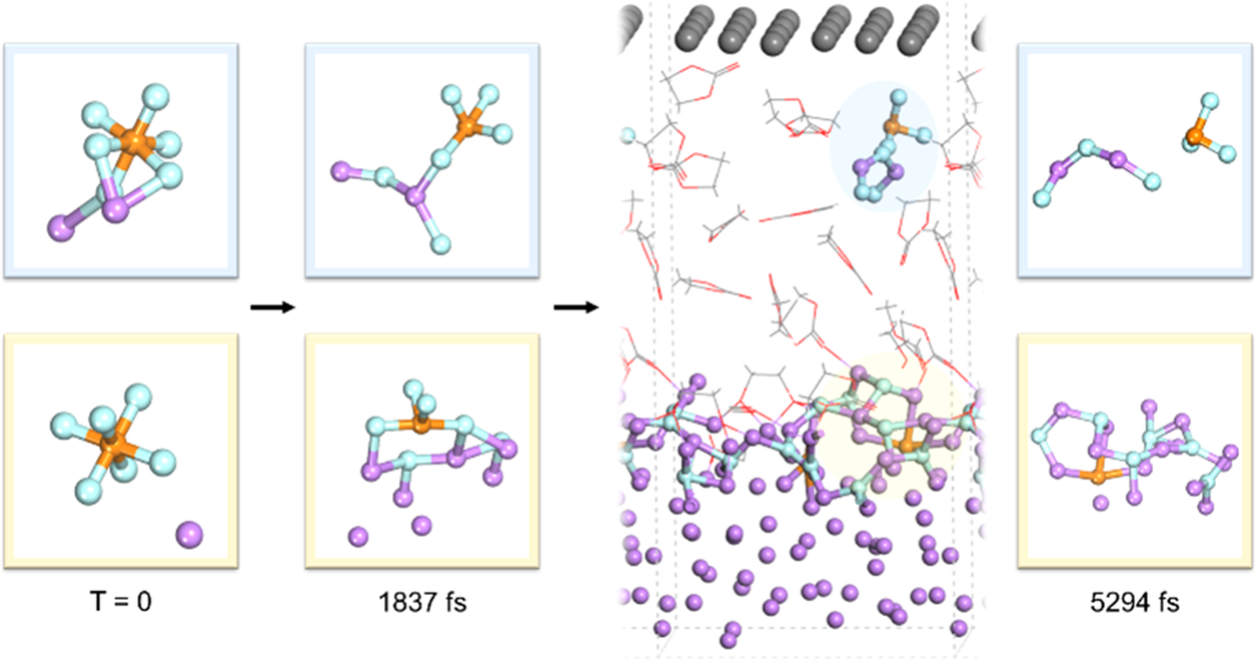
AIMD simulation of LiPF_6_ decomposition on and near lithium metal surface. The initial electrolyte configuration is composed of 3 Li^+^, 3 PF_6_^–^, 18 EC, and 1 VC structures, with a density of 1.3 g/mL. The coordinates of the top-layer He atoms (dark gray) are fixed to refrain electrolyte molecular movement from contacting the lithium layer repeated in the *z*-direction due to periodic boundary conditions. Some cluster structures are rotated for a clear perspective. Color scheme: He (gray ball); C (gray dash); H (white dash); O (red dash); Li (purple ball); P (tan ball); F (cyan ball).

We perform AIMD simulations to reveal the ultrafast decomposition of LiPF_6_ in different electrolyte systems. The initial configuration contained PF_6_^–^ structures with different lithium coordination degrees and distances to the lithium metal surface. The time scale required for salt degradation varies in different solvation environments. In the cyclic carbonate-solvated system (Figure 1), the PF_6_^–^ structures close to the lithium metal surface easily decompose within 2 ps of simulation time. Following such an event, the EC and VC structures disintegrate into carbonyl/carbon monoxide and ethylene di-carbonate (EDC). The solvent degradation pathways have been investigated in many systematic works.^42−45^ In addition to the complete degradation at the electrolyte–metal interface, we also observe the partial dissociation phenomenon of [Li_2_PF_6_]^+^ complex, which is located 15 Å from the lithium metal surface and 8 Å from the top periodic boundary of the *x*–*y* plane in Figure 1 (blue-circled). Such a process is reproducible in other solvent systems, such as DME-DOL. The rearranged structure resembles a seesaw-shaped PF_4_ coordinated with a rhombic Li_2_F_2_ complex. Throughout the P–F cleavage and rearrangement processes, chronological Bader charge analysis (see Figure S1 for details) indicates no charge variation occurring on neighbor electrolyte species or He; only Li and PF_6_ clusters are involved in the charge transport without direct contact. Therefore, we suggest that such charge transfer-driven partial decomposition may be caused by electron tunneling from lithium metal to the dual-lithiated cation.

Moreover, such partial dissociation highly depends on the dual-lithium coordination to hexafluorophosphate and on the cluster charge state. Specifically, in the comparable configuration without extra “free” Li^+^ available in the neighboring region of LiPF_6_ or PF_6_^–^, the partial dissociation does not occur in the corresponding AIMD simulations. With dual lithiation, the P–F cleavage ceases after the [Li_2_PF_6_]^+^ cluster receives an electron from Li metal and becomes negatively charged. We also compute Li^+^ affinity energetics of different electrolyte species (Figure S2) and find that the lithiation free energies for the formation of [Li_2_PF_6_]^+^ and [solvent-Li]^+^ adduct are close to each other. Therefore, the population ratio of [Li_2_PF_6_]^+^ cluster is equivalent to that of the solvated Li^+^ in concentrated electrolytes. The [Li_2_PF_6_]^+^ partial decomposition depicted above should be a non-negligible event during LMB fabrication.

**Figure 2:**
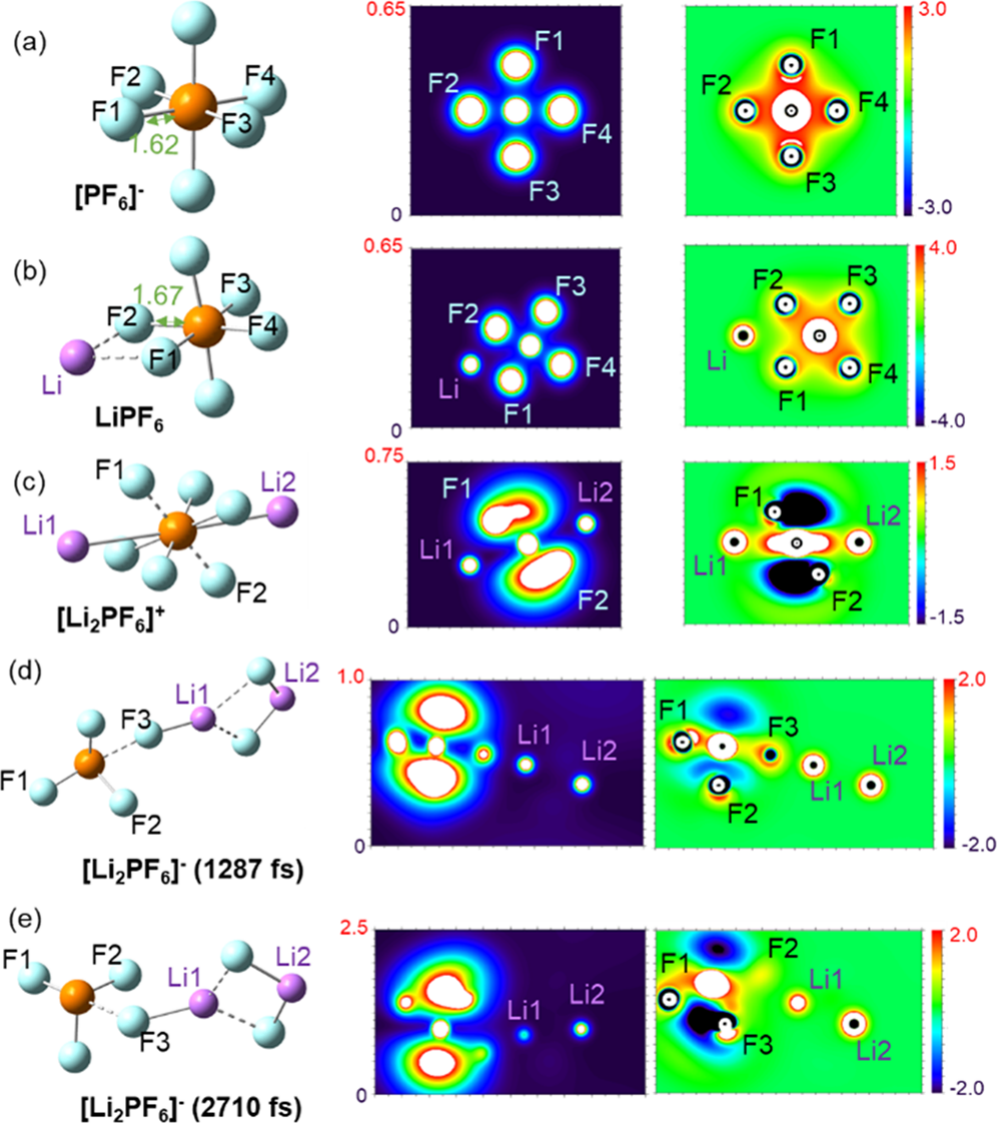
Geometry (left), electron density (middle), and electronic Laplacian (right) analysis of Li_2_PF_6_ clusters, including the optimized geometries of [PF_6_]^−^ (a) LiPF_6_ (b), [Li_2_PF_6_]^+^ (c), as well as the [Li_2_PF_6_]^−^ structures (d, e) retrieved from AIMD simulations.^49^ All of the electronic structure figures are plotted based on the planes determined by labeled atoms. Atomic color coding in structural geometries is identical to Figure 1. The rainbow color scale is labeled on the left (electron density) and right (Laplacian) sides of each diagram, where red indicates positive, and blue indicates zero/negative values.

We further analyze the Li-coordination’s impacts on the electronic structure of [PF_6_]*^x^* species. Previous studies have shown that lithiation reconstructs common electrolyte solvent molecules’ HOMO and LUMO structures.^46,47^ As suggested in Figure 2a,b, P–F bonding has strong covalency in PF_6_^–^ anion and neutral LiPF_6_ molecule, while the bond length is slightly extended due to the lithium coordination. When two lithium atoms are coordinated, some particular P–F bonds demonstrate ionic properties, as the electronic Laplacian planar plot indicates. The P–F distance (dashed bonds in 2d and 2e) also extends from 1.7 to 2.0 Å. Meanwhile, it is interesting to notice that the Li–P interaction shows positive Laplacian in the anionic cluster, suggesting covalent interactions between Li and P atoms, unlike the ionic Li–F cases. Therefore, high-level lithiation destabilizes the electronic configuration in [PF_6_]*^x^* structure.

The semi-equilibrium geometry of [Li_2_PF_6_]*^x^* cluster, as described in Figures 1 and 2, is reproducible in AIMD simulations of both electrolyte systems of cyclic carbonate and ether solvents and is able to maintain its integrity within the feasible simulation time range (see Figures S1 and S3 for additional details). Based on Mulliken charge analysis of the two anionic [Li_2_PF_6_]^−^ structures, the partial charges of Li_2_F_2_ moieties are generally neutral while the rest of the PF_4_ substructures occupy most of the negative charge density (−0.83 and −0.87, respectively). Current computing power limits AIMD from probing the final chemical forms of the partially dissociated [Li_2_PF_6_]*^x^* cluster. Over a longer time scale, such species may either (a) join undissociated PF_6_^–^ anions and serve as ionic carriers in liquid electrolyte or (b) further decompose and become part of SEI. The close–shell [PF_4_]^−^ anion generated from Li1-F3 cleavage has a smaller diameter than the unreacted [PF_6_]^−^ and is thus expected to have a higher diffusion coefficient. From the battery electrolyte and SEI engineering perspective, the PF_6_^–^ partial degradation near the anode material can lead to electrolyte ionic conductivity properties that differentiate from the direct measurement of pure LiPF_6_ solution. Meanwhile, the electrolyte dielectric constant not only impacts the solvation of ionic species but also influences the electron transport rate from the metallic surface, determining the quantities of corresponding products.^25,48^ For instance, it requires less than 1 ps in ether electrolytes (lower ε) to obtain the metastable [Li_2_PF_6_]^−^ structure, while the equivalent process takes more than 2 ps in the carbonate system (higher ε). The Bader charge analysis of both systems (Figure S5) further confirms that such reductive dissociation results in significant charge variations on the lithium metal surface. Therefore, future LMB development could also benefit from the real-time analysis of electrolyte composition in working conditions.

### Lithium Metal Surface Morphology

The lithium surface facet was found to be contributive to the electrolyte carbonate solvent defluorination thermodynamic pathways.^8^ The surface Li–Li distances vary from 3.51 Å (100), 3.04 Å (110), to 4.96 Å (111), depending on the facets. The degradation energy profile of LiPF_6_ also shows a significant mechanistic correlation with the regional metal surface structure.

**Figure 3:**
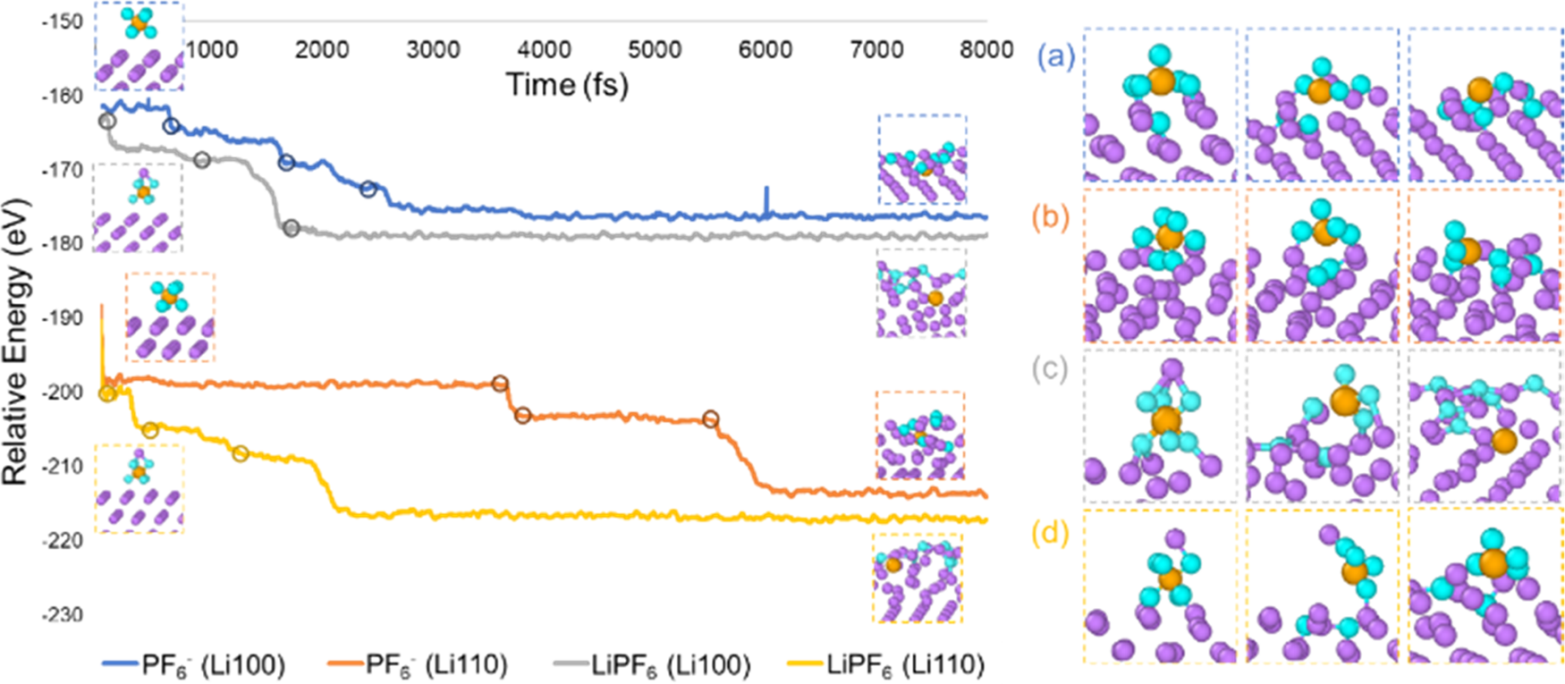
Energy profile of the hexafluorophosphate dissociation on different lithium metal facets retrieved from AIMD simulations. Important events are labeled with (a), (b), (c), and (d). The background charge of configurations (a) and (b) are both −1 e, while panels (c) and (d) are neutral. Atomic color coding is consistent with Figure 1.

We perform AIMD simulations on hexafluorophosphate species with different initial lithiation statuses. Background charge of opposite sign is applied to PF_6_^–^-containing slabs (a) and (b) as no counter ion is included. Bader charge analysis of the initial configurations shows that the PF_6_ clusters in both cases have −1.1 e equivalent charges, while lithium atoms representing bulk structures are generally neutral. Comparing Figure 3a–c,b–d, charge-neutral LiPF_6_ demonstrates faster interfacial adsorption and dissociation than PF_6_^–^ anion in equivalent Li facets. Mulliken charge analysis shows that lithium coordination “pushes” electron density to the fluorine atoms on the opposite side. In other words, the bare fluorine atoms are more electronegative than the Li-coordinated ones in a hexafluorophosphate cluster. The uneven internal charge distribution of LiPF_6_ is mainly responsible for the increased degradation rate.

Based on the time required to reach comparable equilibrium, the hexafluorophosphate dissociation on Li(110) is slightly faster than that on Li(100), which has longer Li–Li distances. The first significant energy relaxation is a consequence of the hexafluorophosphate adsorption to the metal interface. Anionic hexafluorophosphate species can form more than four binding sites with the lithium metal surfaces before dissociation. When PF_6_^–^ approaches the Li(100) surface, five F atoms simultaneously coordinate with neighboring Li atoms in 1 ps. PF_6_^–^ maintains its geometric integrity longer until adsorbing to the Li(110) facet after approximately 3.5 ps. Comparatively, the charge-neutral LiPF_6_ allows three fluorine atoms to bind with interfacial Li atoms upon adsorption. Thus, given the impact of salt concentration and solvent chemistry on the electrolyte structure at the Li metal/electrolyte interface, we expect that the observed significant chemical difference in the interactions of the anion with the surface highlighted here would influence the SEI properties derived from each electrolyte.

The differences in adsorption patterns result in the facet-dependency of fluorophosphate decomposition pathways. In Li(100) configurations, anionic PF_6_^–^ experiences one-by-one sequential P–F bond cleavages (Figure 3a), while two fluorine atoms simultaneously cleave from the LiPF_6_ structure (Figure 3c). A similar two-atom dissociation happens to PF_6_^–^ on Li(110) (Figure 3b), while three fluoride anions leave the phosphorous center of LiPF_6_ (Figure 3d). It is worth noting that at least two Li atoms are coordinated with the dissociated F atom in all of the dissociation events.

Based on our findings, anode metal surface modifications could be a rational strategy in SEI engineering. The Li–Li distances and surface charge distributions profoundly influence the SEI growing rate. Therefore, delicate modifications, including doping and chemical pretreatment, may tune the SEI to ideal structures and functional performance.^50^

Combined with the electronic structure evidence in the Introduction section, we can conclude that hexafluorophosphate degradation requires multiple (at least three) lithium coordinations to fluorine atoms. The semi-covalent Li–P interaction destabilizes P–F bonds and induces further charge transfer from nearby electron-rich lithium atoms. The accumulated negative charges further result in P–F bond cleavages. We compute the energy profile at the molecular level to quantitatively evaluate the impacts of lithiation and solvation in the decomposition pathway.

### Hexafluorophosphate Degradation Pathway and Solvation’s Role in Thermodynamic Profiles

**Figure 4:**
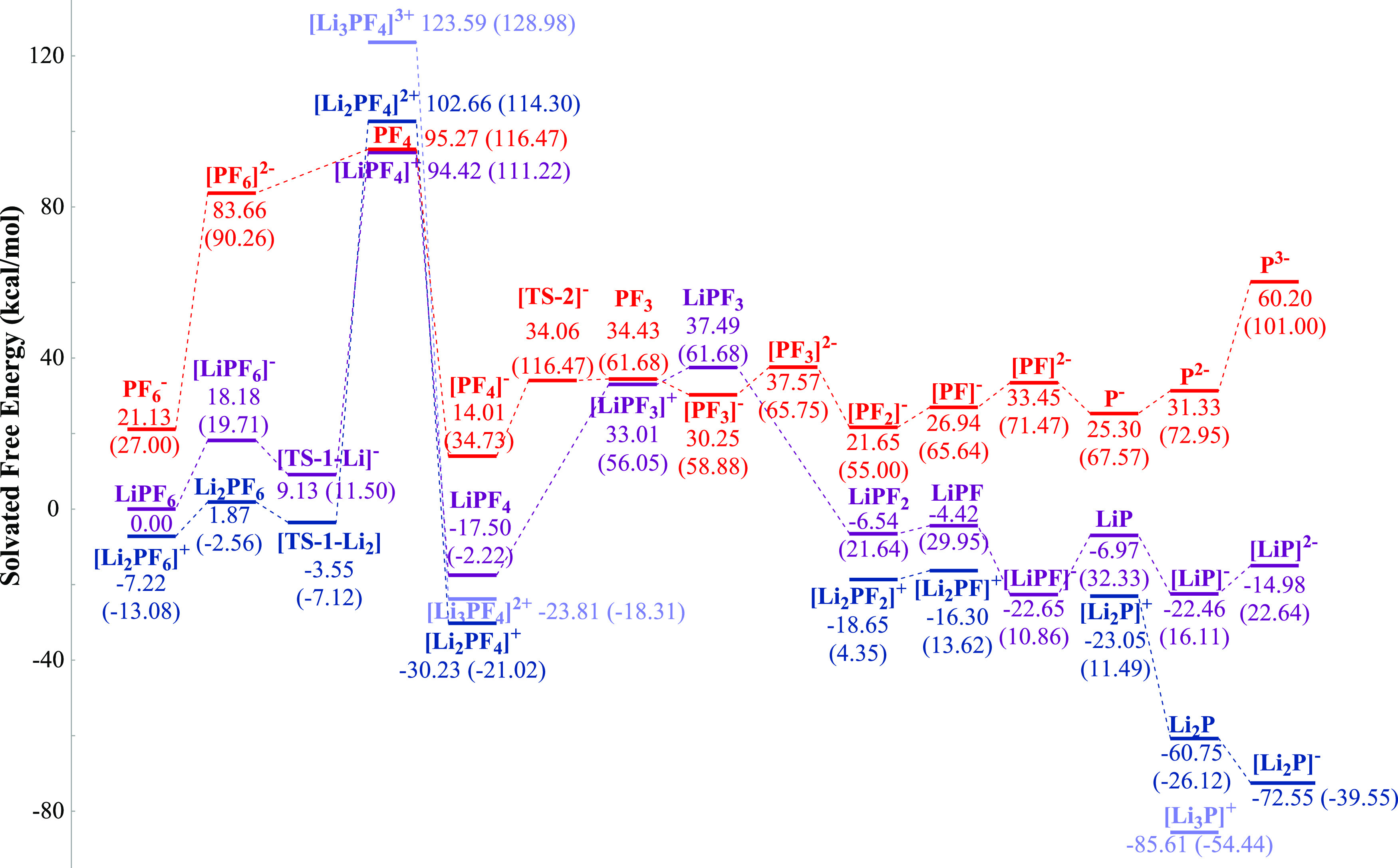
Free energy profile of LiPF_6_ degradation computed in SMD (ε = 7.4). The red, purple, blue, and gray curves stand for nonlithiated, mono-lithiated, bi-lithiated, and tri-lithiated species, respectively. The relative free energies are labeled in kcal/mol, so are the enthalpy values in parenthesis.

To further understand the LiPF_6_ decomposition reaction mechanism in the electrolyte, we investigate the energy profiles in different solvation systems. The Bader charge analysis of key time points in hexafluorophosphate degradation AIMD (Figures S3–S4 in the Supporting Information) demonstrates two necessary steps for P–F bond cleavages: (1) lithium coordination; (2) [PF*_x_*]^*y*−^ cluster receives external electron(s). Based on these criteria, we propose a plausible reaction mechanism that includes electron uptake followed by P–F bond cleavage. We also investigate the lithium bonding’s thermodynamic role in such processes. In addition to the implicit SMD model, which mimics the DME-DOL electrolyte (Figure 4), we also obtain the free energy surface of the identical reaction pathway in higher dielectric constant and in gas-phase conditions shown in Figures S3 in the SI. Such a thermodynamic analysis of the primary SEI fluorination reaction in different solvation environments benefits a quantitative understanding of the reaction pathway and promotes rational electrolyte tuning in future work.

To model the complex solution–metal interfacial reactions, the relative energetics of each species are calculated with respect to LiPF_6_, Li^+^, Li(0), and F^–^ (Figure 4). This method provides rich information in understanding the stepwise energy barriers while being conservative in evaluating the exergonicity and exothermicity, especially when the generated particles embed into the solid surface. The largest free energy uphill is found at the electron-receiving step in forming the closed-shell PF_4_ structures. Lithiation does not show any thermodynamic assistance in this step, while the thermochemistry is particularly favorable in the later reaction stages when more lithium cations bind with the intermediates. This is consistent with the AIMD finding that the dissociation progress is significantly faster after two P–F bonds are cleaved. Figure 4 shows that the nonlithiated pathway is unfeasible in the medium-dielectric environment, and the singly lithiated one is reluctant to proceed. The thermochemistry is generally favorable when at least two lithium atoms are coordinated. Therefore, the lithiation of [PF*_x_*]^*y*−^ intermediate induces charge transfer and plays an assistive role in P–F bond breaking.

We find that the transition states for P–F cleavages do not form significant thermodynamic obstacles; the electron uptake steps hinder the degradation instead. Note that compared with free energy, the enthalpy change in each stage is generally more positive/less negative, indicating that the dissociation progress is primarily entropy-driven. When the decomposition finishes and the initial SEI layer forms, the entropy reaches a local maximum. Besides the energy increase in generating the corresponding open-shell [PF*_x_*]^*y*−^ structures, charge transfer would be more sophisticated after the metallic surface is passivated.

We also assess solvation effects’ role in hexafluorophosphate degradation. In the gas phase, where the dielectric constant is zero, the reaction shows a clear increasing trend in the free energy surface (Figure S6a). Such a pattern is completely inverted in high-dielectric-constant solvation conditions. As shown in Figure S6c, with a solvation environment comparable to the EC–VC electrolyte, the energy barrier for generating PF_4_ is significantly lower than that in DME-DOL. In addition, the following P–F dissociation steps are thermodynamically favorable in all of the lithiation conditions. The free energy uphill of all steps after generating the low-spin PF_4_ species is significantly lower than the corresponding medium-dielectric scenario.

The solvation effects play two significant roles in the initial layer formation reactions of SEI. One is hindering direct electron tunneling between lithiated hexafluorophosphate species and lithium metal, and the other is creating a stabilizing solvation shell for intermediates in salt degradation. Thus, both the ionic conductivities and viscosity are altered together with the SEI formation kinetics and morphology when tuning the LMB liquid electrolyte. In the meantime, because the dissociation reaction rates are strongly correlated with the dielectric environment, people may rationally design the reagents for artificial SEI construction to control the ratios and distributions of different SEI components based on the criteria we present in this work.

## Conclusions

This work showcases the complex chemical interactions among LiPF_6_, electrolyte solvent, and lithium metal. The hexafluorophosphate structures that directly contact the Li metal surface easily experience further lithiation at the electrolyte–lithium–metal interface, where electrons are available for further reduction. The early-stage pathways and reaction rates of such interfacial dissociations are dependent on the regional lithium metal morphology. According to the continuum modeling, strong solvation effects and high lithiation degrees are assistive toward the decomposition thermochemistry. Energy barriers are flatter in a higher dielectric environment. In the solution phase, the electrolyte-dissolved hexafluorophosphate species is partially protected from complete degradation by limited lithiation and charge-transfer rates. Nonetheless, the dual-lithiated species [Li_2_PF_6_]^+^ can still be incompletely reduced via electron tunneling without being adsorbed to the lithium surface. This partial decomposition is caused by elevated ionicity and destabilized electronic configurations of P–F bonding properties in the reduced and multilithiated structures.

The interfacial LiPF_6_ degradation rate is associated with the porosity and density of the SEI initial layer. Techniques, including the surface modification for Li(110) facet exposure and pretreatments using high-dielectric LiPF_6_-containing solutions, would benefit the formation of a dense LiF layer on lithium metal. The detailed mechanistic and thermodynamic information can be incorporated into coarse-grained kMC simulations for the understanding of SEI evolution processes on a larger time and length scale.

## Supporting Information Available

Details of charge distribution, atomic coordinates, energetics, etc. are available in the docx file. This information includes a stepwise free energy table for LiPF_6_ dissociation; chronological Bader charge analysis of the electron-tunneling-induced [Li_2_PF_6_]^+^ partial dissociation; lithium cation affinity energetics; Bader charge analysis of key AIMD frames of DME-DOL-solvated system; chronological Bader charge analysis of [LiPF_6_] and [PF_6_]^−^ interfacial degradations; significant events in forming metastable [Li_2_PF_6_]^−^ structures in EC–VC and DME-DOL electrolytes; free energy profiles of LiPF6 decomposition in different implicit solvation environments; detailed energetics of species involved in LiPF6 decomposition pathways; and Cartesian coordinates of important structures (PDF)

## Abbreviations

SEI: solid electrolyte interphase
LiPF_6_: lithium hexafluorophosphate
LMB: lithium metal battery
LiFSI: bis(fluorosulfonyl)imide
FEC: fluoroethylene carbonate
AIMD: ab initio molecular dynamics
DME: dimethoxyethane
DOL: 1,3-dioxolane
ε: dielectric constants
EC: ethylene carbonate
PC: propylene carbonate
VC: vinylene carbonate
MD: molecular dynamics
kMC: kinetic Monte Carlo
EDC: ethylene di-carbonate
SMD: solvation model based on density
PBE: Perdew–Burke–Ernzerhof
GGA: generalized gradient approximation
PAW: projector augmented wave
