# Supplementary material for: Inorganic Solid Electrolyte Interphase Engineering Rationales Inspired by Hexafluorophosphate Decomposition Mechanisms
Source: J Phys Chem C Nanomater Interfaces. 2023 Jan 23;127(4):1744–51. doi: 10.1021/acs.jpcc.2c07838 (PMC10848255; doi:10.1021/acs.jpcc.2c07838)
Supplement: Supplementary file 1 — jp2c07838_si_001.pdf [file jp2c07838_si_001.pdf]

Supporting Information for
Inorganic Solid Electrolyte Interphase Engineering Rationales Inspired by Hexafluorophosphate Decomposition Mechanisms

Dacheng Kuai[1,2] and Perla B. Balbuena[1,2,3]*.

[1]Department of Chemical Engineering, Texas A&M University, College Station, Texas 77843, USA
[2]Department of Chemistry, Texas A&M University, College Station, Texas 77843, USA
[3]Department of Materials Science and Engineering, Texas A&M University, College Station, Texas 77843, USA.

Table of Contents

Stepwise free energy table for $LiPF_6$ dissociation **S2**

Chronological Bader charge analysis of the electron-tunneling induced $[Li_2PF_6]^+$ partial dissociation **S2**

Lithium cation affinity energetics **S3**

Bader charge analysis of key AIMD frames of DME-DOL-solvated system **S3**

Chronological Bader charge analysis of $[LiPF_6]$ and $[PF_6]^-$ interfacial degradations **S4**

Significant events in forming metastable $[Li_2PF_6]^-$ structures in EC-VC and DME-DOL electrolytes **S5**

Free energy profiles of $LiPF_6$ decomposition in different implicit solvation environments **S6**

Detailed energetics of species involved in $LiPF_6$ decomposition pathway **S8**

Cartesian coordinates of important structures **S11**

Table S1. Stepwise free energy in generating (Li)PF_x^y intermediates. Multiplicity values are marked in parentheses for each high-spin state species. All structures were optimized at B3PW91/6-311G(3df) level of theory, and energy values are in unit of kcal/mol.

	High Multiplicity				Low Multiplicity (singlet)			
	$PF_x^- + Li^0 \rightarrow LiF + PF_{x-1}^-$		$LiPF_x + Li^0 \rightarrow LiF + LiPF_{x-1}$		$PF_x^{n-} + Li^+ + (e^-) \rightarrow LiF + PF_{x-1}^{m-}$		$LiPF_x^{n-} + Li^+ + (e^-) \rightarrow LiF + LiPF_{x-1}^{m-}$	
x	ΔG	Species	ΔG	Species	ΔG	Product	ΔG	Product
6	**+17.67**	PF_5^- (2)	-7.96	$LiPF_5$ (2)	**+13.79**	PF_5	**+37.11**	$LiPF_5^+$ (-Li)
5	-46.83	**PF_4^- (1)**	-82.92 (eq); -62.69 (ax)	$LiPF_4$ (1)	-123.37	PF_4^- (-F)	-208.41 (eq); -188.17 (ax)	$LiPF_4$
4	-73.16	PF_3^- (2)	-11.70	$LiPF_3$ (2)	-90.36	PF_3	-11.96	$LiPF_3^+$(-F)
3	-35.00	**PF_2^- (1)**	-70.12	$LiPF_2$ (1)	-98.21	PF_2^-	-150.26	$LiPF_2$
2	-88.86	PF^- (2)	-56.67	LiPF (2)	-189.43	PF^{2-}	-152.42	$LiPF^-$
1	-52.53	P^- (3)	-31.71	LiP (3)	-157.76	P^{3-}	-175.78	LiP^{2-}

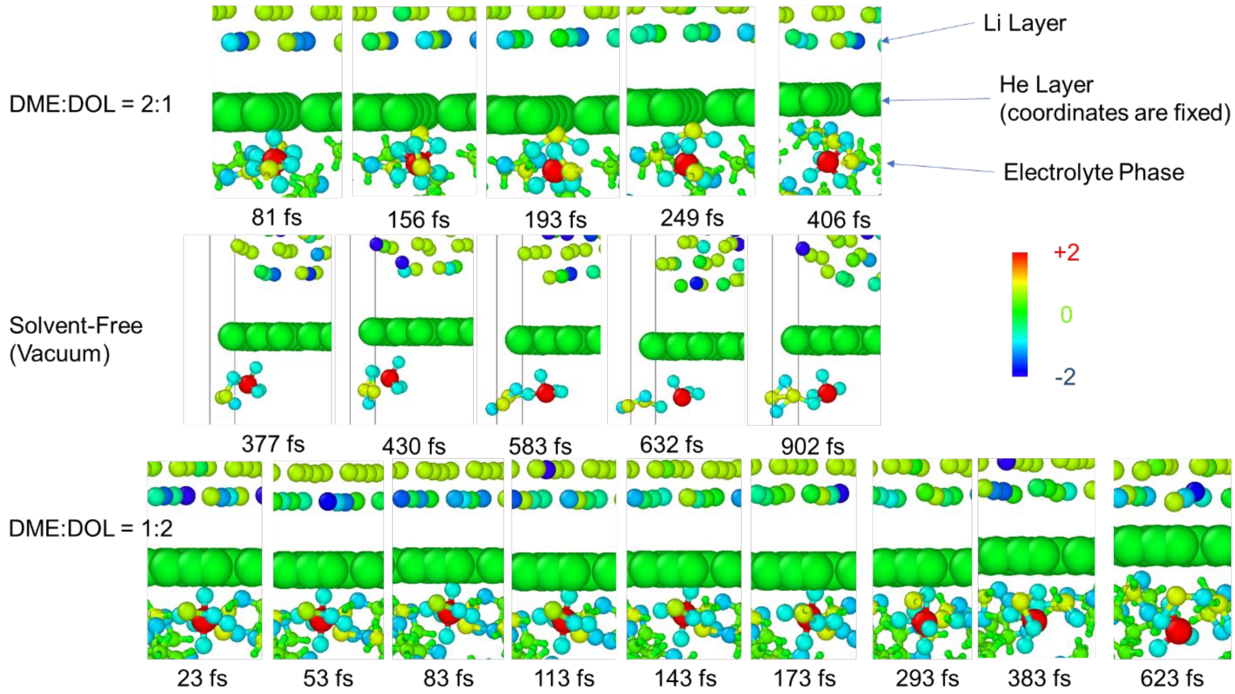

Figure S1. Chronological Bader charge analysis of the electron-tunneling induced $[Li_2PF_6]^+$ partial dissociation.

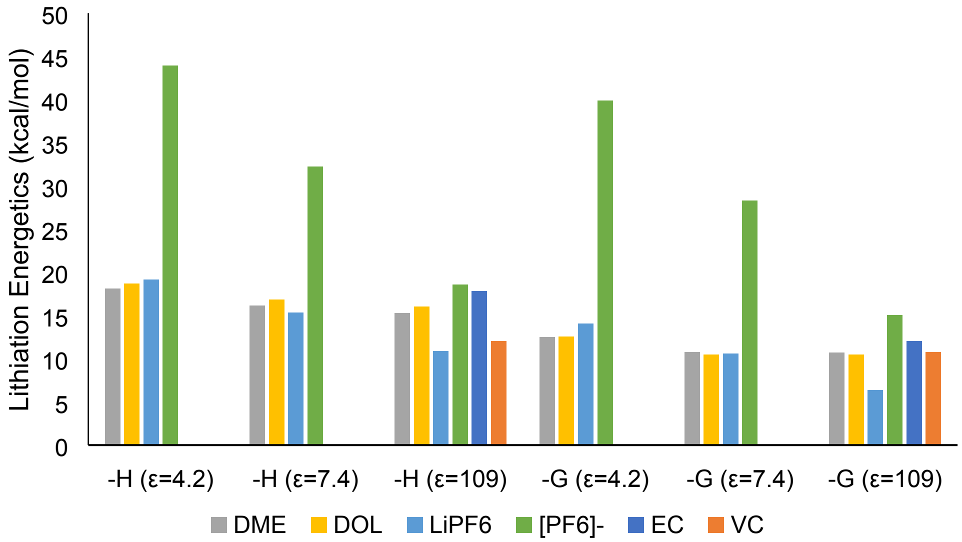

Figure S2. Lithium cation affinity energetics analyzed in different dielectric environments. All structures were optimized at B3PW6/6-311G(3dp) level of theory.

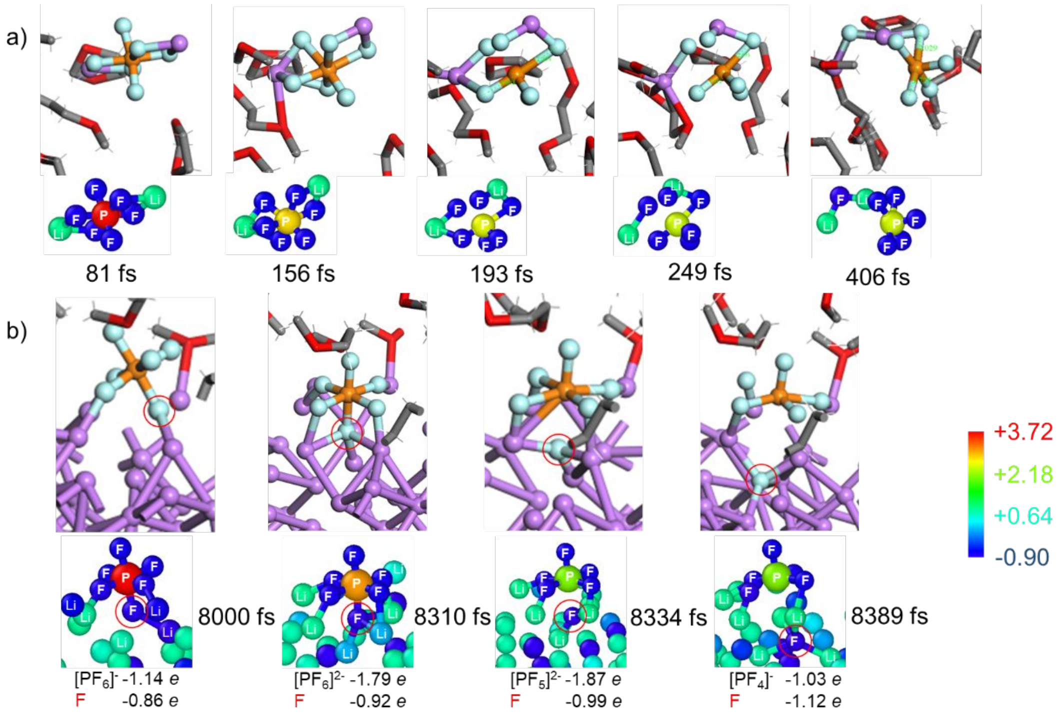

Figure S3. Bader charge analysis of key AIMD frames of DME-DOL-solvated $[Li_2PF_6]^+$ (a) and $[PF_6]^-$ degradation at Li-electrolyte interface (b). Charge values beneath the snapshots are the equivalent fragment charge and the atomic charge for circled F atom. Atomic color coding is identical to Figure 1.

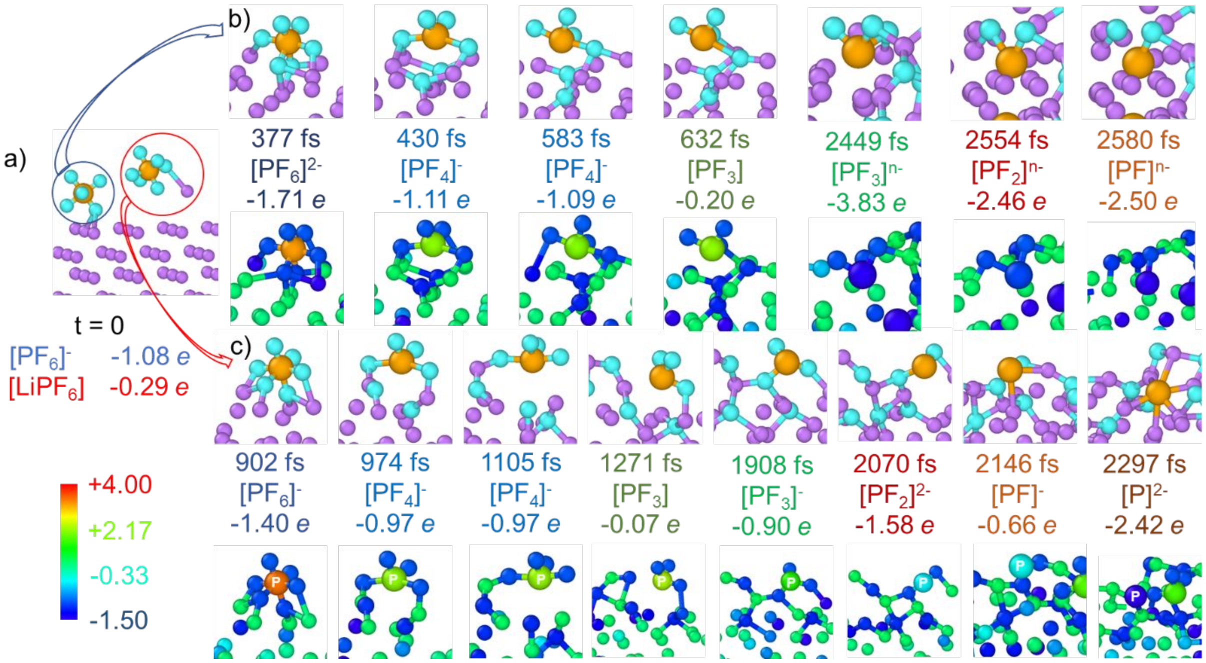

Figure S4. Chronological Bader charge analysis of key AIMD frames in interfacial degradation of [$LiPF_6$] (b) and $[PF_6]^-$ (c). Values labeled beneath each snapshot include time point in fs, $[PF_x]^y$ structure, and the equivalent fragment charge. Atomic color coding is the same as Figure 1.

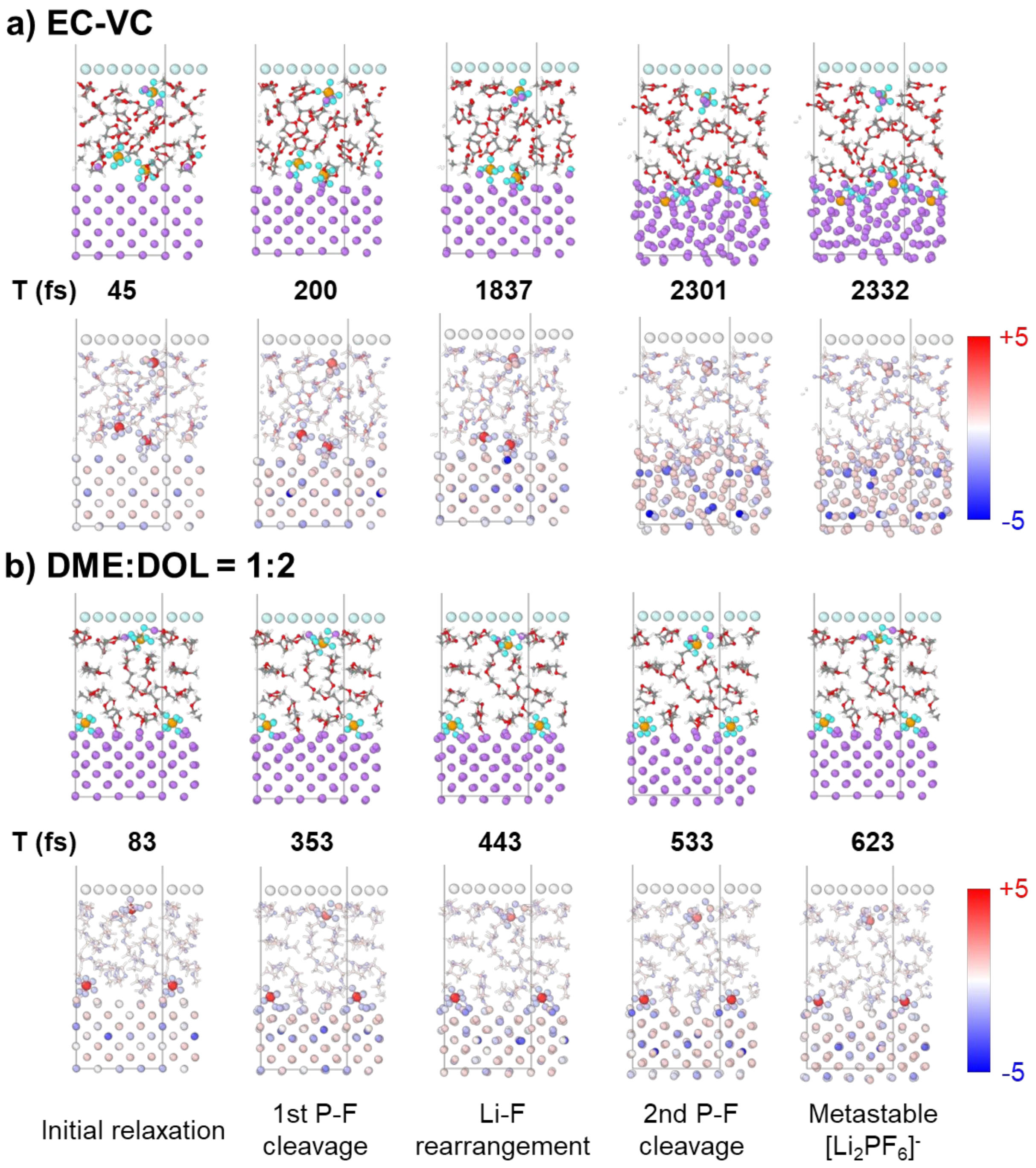

Figure S5. Significant events in forming metastable $[Li_2PF_6]^-$ structures in EC-VC (a) and DME-DOL (b) electrolyte systems as well as their charge variations. The regular color coding for the top configurations is consistent with Figure 1, and the lower charge distributions have symmetric color bar indicated on the right side.

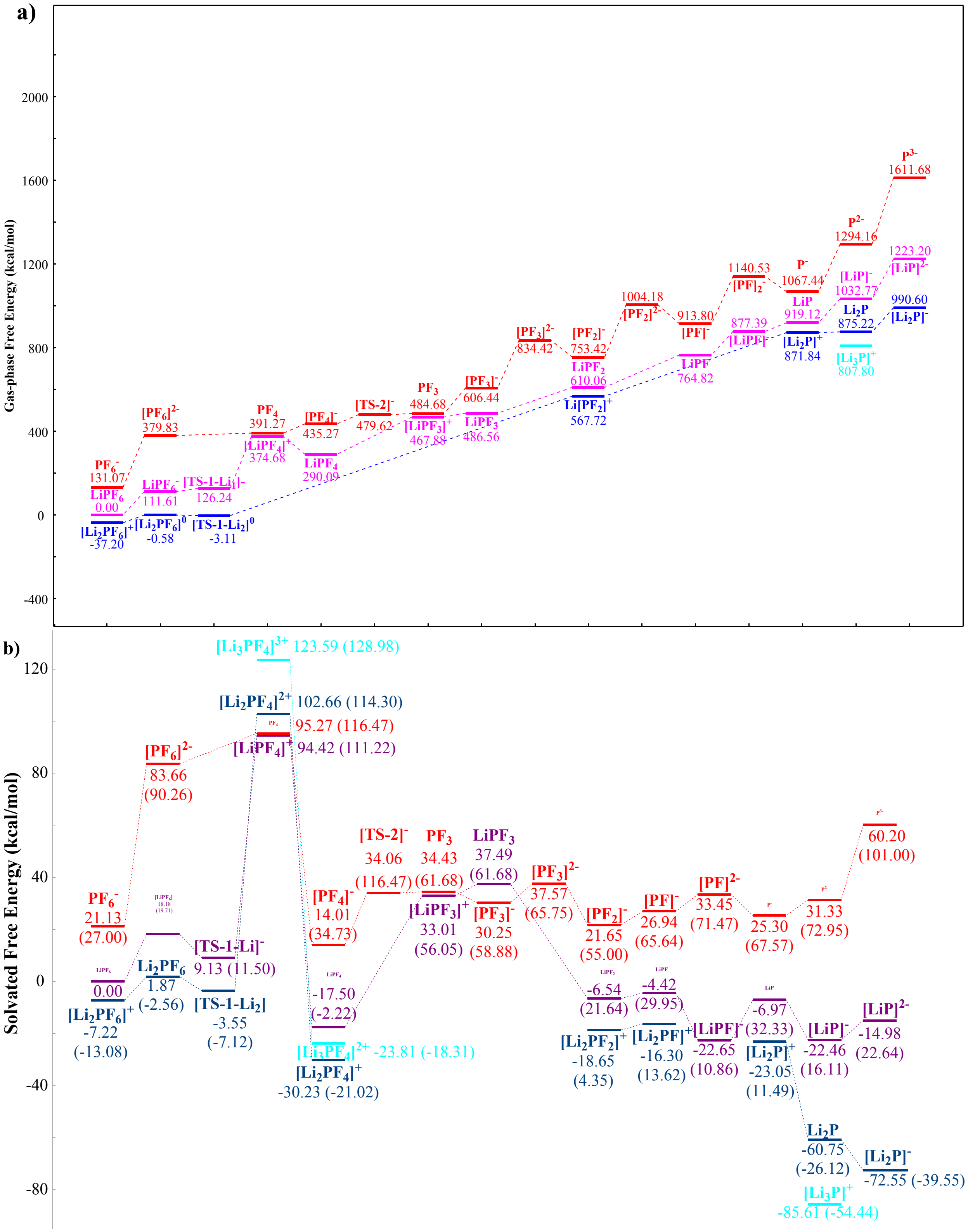
a)
Gas-phase Free Energy (kcal/mol)
2000
1600
1200
800
400
0
-400
PF6-
131.07
LiPF6
0.00
[Li2PF6]+
-37.20
[PF6]2-
379.83
LiPF6-
111.61
[Li2PF6]0
-0.58
[TS-1-Li]-
126.24
[TS-1-Li2]0
-3.11
PF4
391.27
[LiPF4]+
374.68
[PF4]-
435.27
LiPF4
290.09
[TS-2]-
479.62
PF3
484.68
[LiPF3]+
467.88
[PF3]-
606.44
LiPF3
486.56
[PF3]2-
834.42
[PF2]-
753.42
LiPF2
610.06
Li[PF2]+
567.72
1004.18
[PF2]2-
913.80
[PF]-
LiPF
764.82
1140.53
[PF]2-
877.39
[LiPF]-
P-
1067.44
LiP
919.12
[Li2P]+
871.84
P2-
1294.16
[LiP]-
1032.77
Li2P
875.22
[Li3P]+
807.80
P3-
1611.68
1223.20
[LiP]2-
990.60
[Li2P]-
b)
Solvated Free Energy (kcal/mol)
120
80
40
0
-40
-80
[Li3PF4]3+ 123.59 (128.98)
[Li2PF4]2+ 102.66 (114.30)
PF4 95.27 (116.47)
[LiPF4]+ 94.42 (111.22)
[PF6]2-
83.66
(90.26)
PF6-
21.13
(27.00)
LiPF6
0.00
[Li2PF6]+
-7.22
(-13.08)
[LiPF6]-
18.18
(19.71)
Li2PF6
1.87
(-2.56)
[TS-1-Li]-
9.13 (11.50)
[TS-1-Li2]
-3.55
(-7.12)
[PF4]-
14.01
(34.73)
LiPF4
-17.50
(-2.22)
[Li3PF4]2+ -23.81 (-18.31)
[Li2PF4]+
-30.23 (-21.02)
[TS-2]-
34.06
(116.47)
PF3
34.43
(61.68)
[LiPF3]+
33.01
(56.05)
LiPF3
37.49
(61.68)
[PF3]-
30.25
(58.88)
[PF3]2-
37.57
(65.75)
[PF2]-
21.65
(55.00)
LiPF2
-6.54
(21.64)
[Li2PF2]+
-18.65
(4.35)
[PF]-
26.94
(65.64)
LiPF
-4.42
(29.95)
[Li2PF]+
-16.30
(13.62)
[PF]2-
33.45
(71.47)
[LiPF]-
-22.65
(10.86)
P-
25.30
(67.57)
LiP
-6.97
(32.33)
[Li2P]+
-23.05
(11.49)
P2-
31.33
(72.95)
[LiP]-
-22.46
(16.11)
P3-
60.20
(101.00)
[LiP]2-
-14.98
(22.64)
Li2P
-60.75
(-26.12)
[Li2P]-
-72.55 (-39.55)
[Li3P]+
-85.61 (-54.44)

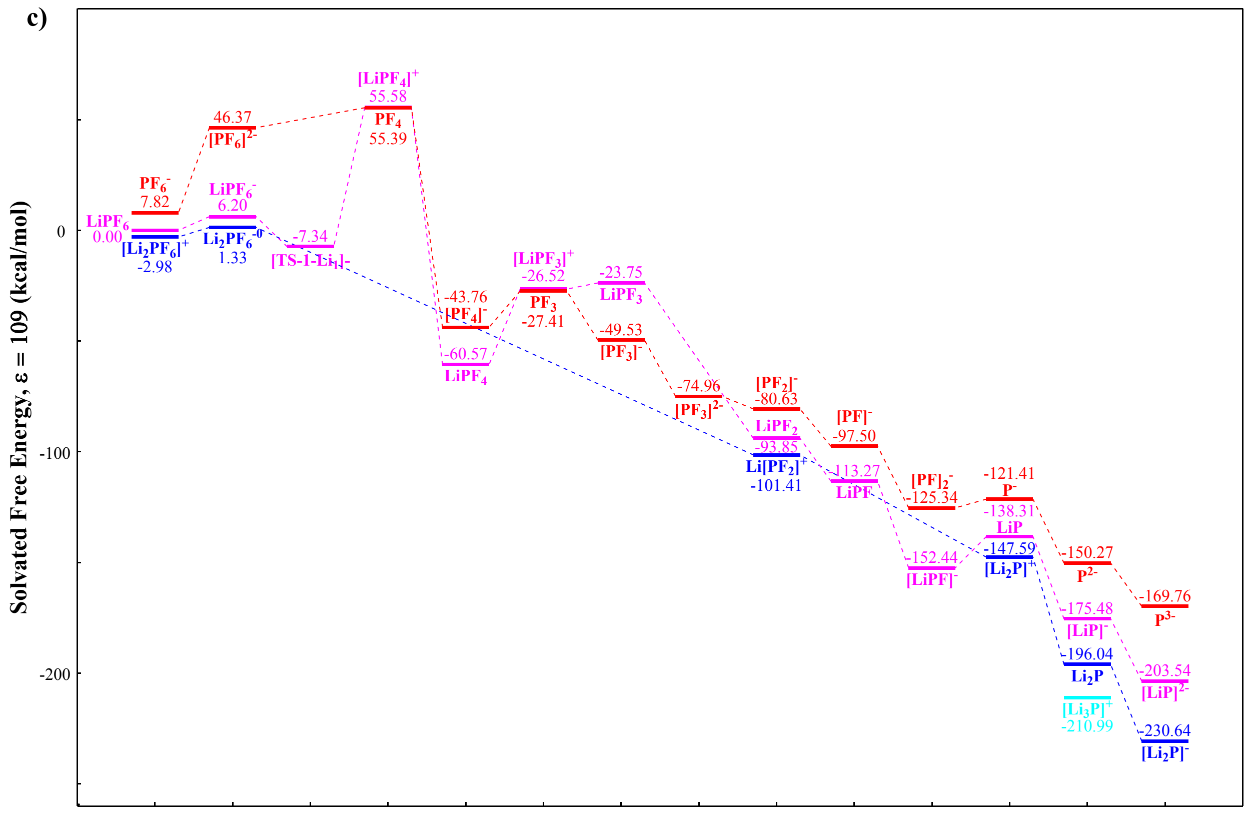

Figure S6. Free energy profiles of $LiPF_6$ decomposition in gas-phase (a), solvated conditions with dielectric constants close to DME-DOL (b) and EC-VC (c) environments. The energy surface for the non-lithiated pathway is colored in red, mono-lithiated in purple, di-lithiated in blue, and tri-lithiated in cyan. All intermediate structures were optimized at the M06-2X/aug-cc-pVDZ level of theory.

Table S2. Gas-phase energies (Hartree), entropy (in cal·mol-1·K^{-1}) and the corresponding charge/multiplicity.

Structure	C/M	Potential Energy	Enthalpy	Entropy	G, 1 atm	G, 1 M
F^-	-1,1	-99.8232	-99.8208	34.7670	-99.8374	-99.8343
Li^+	1,1	-7.2823	-7.2800	31.7980	-7.2951	-7.2920
Li	0,2	-7.4810	-7.4787	33.1750	-7.4944	-7.4914
$[Li_2PF_6]^+$	1,1	-955.4251	-955.3918	92.2780	-955.4356	-955.4326
Li_2PF_6	0,2	-955.5613	-955.5286	101.1140	-955.5766	-955.5736
$LiPF_6$	0,1	-948.0752	-948.0450	82.6920	-948.0843	-948.0813
$[LiPF_6]^-$	-1,2	-948.0941	-948.0645	87.0090	-948.1058	-948.1028
PF_6^-	-1,1	-940.5725	-940.5461	78.4930	-940.5834	-940.5804
$[PF_6]^{2-}$	-2,2	-940.3745	-940.3484	79.8370	-940.3863	-940.3833
**[TS-1-Li]**$^-$	-1,2	-948.0651	-948.0391	91.3110	-948.0825	-948.0795
**[TS-1-Li2]**	0,2	-955.5660	-955.5362	93.4550	-955.5806	-955.5776
$[Li_2PF_4]^{2+}$	2,2	-755.2113	-755.1880	96.3980	-755.2338	-755.2308
$[Li_2PF_4]^+$	1,1	-755.6950	-755.6718	85.9480	-755.7127	-755.7097
$[LiPF_4]$+	1,2	-747.9977	-747.9769	86.1750	-748.0179	-748.0149
$LiPF_4$	0,1	-748.3336	-748.3135	81.1380	-748.3521	-748.3490
PF_4	0,2	-740.6817	-740.6641	74.3230	-740.6994	-740.6964
$[PF_4]^-$	-1,1	-740.8097	-740.7935	74.0790	-740.8287	-740.8256
**[TS-2]**$^-$	-1,1	-740.7328	-740.7177	84.8860	-740.7580	-740.7550
$[LiPF_3]^+$	1,1	-648.2125	-648.1962	80.4820	-648.2344	-648.2314
$LiPF_3$	0,2	-648.3814	-648.3666	78.6360	-648.4040	-648.4010
PF_3	0,1	-640.8967	-640.8833	67.9380	-640.9156	-640.9126
$[PF_3]^-$	-1,2	-640.8975	-640.8859	73.6590	-640.9209	-640.9179
$[PF_3]^{2-}$	-2,1	-640.7330	-640.7222	73.1300	-640.7569	-640.7539
$[Li_2PF_2]^+$	1,1	-555.9100	-555.8951	77.1280	-555.9317	-555.9287
$LiPF_2$	0,1	-548.5509	-548.5389	70.0090	-548.5722	-548.5692
$[PF_2]^-$	-1,1	-541.0303	-541.0220	62.5010	-541.0517	-541.0487
LiPF	0,2	-448.6684	-448.6604	63.5430	-448.6906	-448.6876
$[LiPF]^-$	-1,1	-448.6888	-448.6808	62.5590	-448.7106	-448.7075
$[PF]^-$	-1,2	-441.1407	-441.1358	53.4240	-441.1611	-441.1581
$[PF]^{2-}$	-2,1	-440.9790	-440.9743	52.3800	-440.9992	-440.9962

$[Li_3P]^+$	1,2	-363.7456	-363.7349	74.9800	-363.7706	-363.7675
$[Li_2P]^+$	1,3	-356.1568	-356.1496	57.9870	-356.1771	-356.1741
Li_2P	0,2	-356.3481	-356.3405	64.2710	-356.3711	-356.3681
$[Li_2P]$-	-1,1	-356.3647	-356.3571	61.9530	-356.3866	-356.3835
LiP	0,3	-348.7890	-348.7843	53.4830	-348.8097	-348.8067
$[LiP]^-$	-1,2	-348.8076	-348.8030	52.6510	-348.8280	-348.8250
$[LiP]^{2-}$	-2,1	-348.7043	-348.6996	51.0860	-348.7239	-348.7208
P^-	-1,3	-341.2654	-341.2631	38.4080	-341.2813	-341.2783
P^{2-}	-2,2	-341.1039	-341.1015	37.6020	-341.1194	-341.1164
P^{3-}	-3,1	-340.7979	-340.7955	36.2240	-340.8127	-340.8097

In the "G 1 M" column, the "1 M" refers to free energies adjusted for a standard state of 1 M instead of the default standard state of 1 atm by adding 0.001987*298.15*ln(24.5)/627.509.

Table S3. Energies (Hartree), entropy (in cal·mol-1·K^{-1}) and the corresponding charge/multiplicity of structures computed with SMD model comparable to the DME-DOL solvation environment (ε=7.4).

Structure	C/M	Potential Energy	Enthalpy	Entropy	G, 1 atm	G, 1 M
F^-	-1,1	-99.94588	-99.94352	34.76700	-99.96004	-99.95702
Li^+	1,1	-7.40675	-7.40439	31.79800	-7.41950	-7.41648
Li	0,2	-7.48247	-7.48011	33.17500	-7.49587	-7.49285
$[Li_2PF_6]^+$	1,1	-955.52444	-955.49273	90.04000	-955.53551	-955.53249
Li_2PF_6	0,2	-955.58407	-955.55168	96.23800	-955.59740	-955.59438
$LiPF_6$	0,1	-948.09715	-948.06750	84.25700	-948.10753	-948.10451
$[LiPF_6]^-$	-1,2	-948.14100	-948.11180	90.79200	-948.15494	-948.15192
PF_6^-	-1,1	-940.64629	-940.62008	78.47900	-940.65737	-940.65435
$[PF_6]^{2-}$	-2,2	-940.61952	-940.59498	82.31100	-940.63409	-940.63107
**[TS-1-Li]$^-$**	-1,2	-948.15003	-948.12489	93.55600	-948.16935	-948.16633
**[TS-1-Li2]**	0,2	-955.58754	-955.55895	99.10800	-955.60603	-955.60301
$[Li_3PF_4]^{3+}$	3,2	-762.88607	-762.85942	97.77300	-762.90588	-762.90286
$[Li_3PF_4]^{2+}$	2,1	-763.19550	-763.16986	99.52700	-763.21715	-763.21413
$[Li_2PF_4]^{2+}$	2,2	-755.50173	-755.47841	93.32400	-755.52275	-755.51973
$[Li_2PF_4]^+$	1,1	-755.79244	-755.76979	86.51200	-755.81089	-755.80787
$[LiPF_4]$+	1,2	-748.09932	-748.07894	85.14000	-748.11939	-748.11637

$LiPF_4$	0,1	-748.35497	-748.33544	81.44700	-748.37413	-748.37111
PF_4	0,2	-740.68354	-740.66618	74.48100	-740.70157	-740.69855
$[PF_4]^-$	-1,1	-740.88815	-740.87216	74.25200	-740.90743	-740.90441
**$[TS\text{-}2]^-$**	-1,1	-740.85464	-740.84013	74.40600	-740.87548	-740.87246
$[LiPF_3]^+$	1,1	-648.31520	-648.29905	79.04000	-648.33661	-648.33359
$LiPF_3$	0,2	-648.38172	-648.36580	84.29600	-648.40585	-648.40283
PF_3	0,1	-640.89870	-640.88552	68.07600	-640.91787	-640.91485
$[PF_3]^-$	-1,2	-640.97727	-640.96587	73.73500	-641.00090	-640.99788
$[PF_3]^{2-}$	-2,1	-641.04136	-641.03064	73.60000	-641.06561	-641.06259
$[Li_2PF_2]^+$	1,1	-556.00841	-555.99376	78.69200	-556.03115	-556.02813
$LiPF_2$	0,1	-548.57335	-548.56180	70.63800	-548.59536	-548.59234
$[PF_2]^-$	-1,1	-541.11244	-541.10425	62.55000	-541.13397	-541.13095
$[LiPF]^+$	1,2	-456.12183	-456.11118	74.88900	-456.14676	-456.14374
LiPF	0,2	-448.68849	-448.68076	64.37100	-448.71134	-448.70832
$[LiPF]^-$	-1,1	-448.79465	-448.78691	62.84000	-448.81676	-448.81374
$[PF]^-$	-1,2	-441.22442	-441.21949	53.45500	-441.24489	-441.24187
$[PF]^{2-}$	-2,1	-441.29061	-441.28592	52.54200	-441.31088	-441.30786
$[Li_3P]^+$	1,2	-363.84234	-363.83195	78.87600	-363.86942	-363.86640
$[Li_2P]^+$	1,3	-356.25391	-356.24677	63.35700	-356.27688	-356.27386
Li_2P	0,2	-356.38974	-356.38243	65.03300	-356.41333	-356.41031
$[Li_2P]$-	-1,1	-356.48719	-356.47954	60.94200	-356.50850	-356.50548
LiP	0,3	-348.81370	-348.80916	53.88900	-348.83476	-348.83174
$[LiP]^-$	-1,2	-348.91534	-348.91074	52.77300	-348.93582	-348.93280
$[LiP]^{2-}$	-2,1	-348.98082	-348.97605	50.99000	-349.00027	-348.99725
P^-	-1,3	-341.35097	-341.34861	38.40800	-341.36686	-341.36384
P^{2-}	-2,2	-341.41811	-341.41575	37.60200	-341.43362	-341.43060
P^{3-}	-3,1	-341.44914	-341.44678	36.22400	-341.46399	-341.46097

Table S4. Energies (Hartree), entropy (in cal·mol-1·K^{-1}) and the corresponding charge/multiplicity of structures computed with SMD model comparable to the EC-VC solvation environment (ε=109).

Structure	C/M	Potential Energy	Enthalpy	Entropy	G, 1 atm	G, 1 M
F^-	-1,1	-99.96287	-99.96051	34.76700	-99.97702	-99.97400

Li^+	1,1	-7.42352	-7.42116	31.79800	-7.43627	-7.43325
Li	0,2	-7.48147	-7.47911	33.17500	-7.49487	-7.49185
$[Li_2PF_6]^+$	1,1	-955.53768	-955.50609	90.54900	-955.54911	-955.54609
Li_2PF_6	0,2	-955.58663	-955.55435	97.81500	-955.60083	-955.59781
$LiPF_6$	0,1	-948.09982	-948.07039	85.69400	-948.11111	-948.10809
$[LiPF_6]^-$	-1,2	-948.14575	-948.11661	90.96700	-948.15983	-948.15681
PF_6^-	-1,1	-940.65422	-940.62808	78.53800	-940.66540	-940.66238
$[PF_6]^{2-}$	-2,2	-940.64993	-940.62422	80.70100	-940.66256	-940.65954
**[TS-1-Li]$^-$**	-1,2	-948.16135	-948.13642	94.67000	-948.18140	-948.17838
**[TS-1-Li2]**	-1,2	-948.16292	-948.13781	95.24800	-948.18307	-948.18005
$[Li_2PF_4]^{2+}$	2,2	-755.54166	-755.51840	93.61200	-755.56287	-755.55985
$[Li_2PF_4]^+$	1,1	-755.80518	-755.78266	87.58200	-755.82427	-755.82125
$[LiPF_4]$+	1,2	-748.11356	-748.09322	83.99400	-748.13312	-748.13010
$LiPF_4$	0,1	-748.35756	-748.33818	81.29700	-748.37681	-748.37379
PF_4	0,2	-740.68207	-740.66477	74.52300	-740.70017	-740.69715
$[PF_4]^-$	-1,1	-740.89740	-740.88147	74.31100	-740.91678	-740.91376
**[TS-2]$^-$**	-1,1	-740.86090	-740.84589	84.34500	-740.88596	-740.88294
$[LiPF_3]^+$	1,1	-648.32874	-648.31361	73.53600	-648.34855	-648.34553
$LiPF_3$	0,2	-648.38044	-648.36449	80.49100	-648.40274	-648.39972
PF_3	0,1	-640.89749	-640.88437	68.09100	-640.91672	-640.91370
$[PF_3]^-$	-1,2	-640.98684	-640.97548	73.85500	-641.01057	-641.00755
$[PF_3]^{2-}$	-2,1	-641.08543	-641.07471	73.60600	-641.10968	-641.10666
$[Li_2PF_2]^+$	1,1	-556.02114	-556.00659	79.43100	-556.04433	-556.04131
$LiPF_2$	0,1	-548.57675	-548.56529	71.01300	-548.59903	-548.59601
$[PF_2]^-$	-1,1	-541.12316	-541.11499	62.56700	-541.14472	-541.14170
LiPF	0,2	-448.69159	-448.68390	64.56900	-448.71457	-448.71155
$[LiPF]^-$	-1,1	-448.81324	-448.80561	63.10200	-448.83559	-448.83257
$[PF]^-$	-1,2	-441.23572	-441.23079	53.46100	-441.25619	-441.25317
$[PF]^{2-}$	-2,1	-441.33888	-441.33419	52.53600	-441.35915	-441.35613
$[Li_3P]^+$	1,2	-363.85504	-363.84661	70.26600	-363.88000	-363.87698
$[Li_2P]^+$	1,3	-356.26620	-356.25907	59.03500	-356.28712	-356.28410
Li_2P	0,2	-356.39849	-356.39130	66.55500	-356.42293	-356.41991
$[Li_2P]$-	-1,1	-356.51456	-356.50714	62.14700	-356.53666	-356.53364
LiP	0,3	-348.81795	-348.81343	53.97700	-348.83908	-348.83606
$[LiP]^-$	-1,2	-348.93630	-348.93174	52.94300	-348.95690	-348.95388

$[LiP]^{2-}$	-2,1	-349.04044	-349.03584	51.32400	-349.06022	-349.05720
P^{-}	-1,3	-341.36300	-341.36064	38.40800	-341.37889	-341.37587
P^{2-}	-2,2	-341.46797	-341.46561	37.60200	-341.48348	-341.48046
P^{3-}	-3,1	-341.55829	-341.55593	36.22400	-341.57314	-341.57012

*Coordinates for each structure are available upon request.

Atomic coordinates of structures in Figure 2

$[PF_6]^-$

P	-0.00007200	0.00008000	-0.00006100
F	1.23702000	0.20793900	1.05933300
F	0.08555800	1.58740000	-0.41177900
F	-1.07620100	0.36501000	1.18530900
F	-1.23753600	-0.20803800	-1.05907700
F	1.07657000	-0.36510500	-1.18446800
F	-0.08529400	-1.58733900	0.41078300

$LiPF_6$

P	0.16275524	-0.00002375	0.00000983
F	-1.06476980	1.13473210	-0.00339129
F	-1.06497294	-1.13460335	0.00282717
F	0.10956443	0.00453924	1.60026504
F	1.25226316	-1.15856943	0.00334507
F	0.11014064	-0.00452595	-1.59999082
F	1.25244157	1.15842433	-0.00308527
Li	-2.59777734	0.00012792	0.00004118

$[Li_2PF_6]^+$

P	0.00124200	0.00194400	0.00172600

F	0.98129600	0.90004500	0.95566100
F	0.96721600	0.39568600	-1.25892800
F	-0.99540800	1.27572400	-0.28491900
F	-0.97971400	-0.89841900	-0.95686200
F	0.98851000	-1.27553200	0.28482900
F	-0.96506900	-0.39193600	1.26282000
Li	2.52102300	-0.00941000	-0.01061900
Li	-2.51772400	-0.01701600	-0.00581300

$[Li_2PF_6]^-$ (1287 fs)

Li	15.46930000	9.50657000	24.97150000
Li	13.13300000	8.52299000	25.03890000
P	9.79060000	6.57300000	25.05000000
F	14.73180000	9.15926000	26.46460000
F	11.51660000	7.63403000	25.24280000
F	10.77540000	5.26806000	24.82870000
F	9.72398000	6.64579000	26.67090000
F	14.49040000	8.96883000	23.63290000
F	8.44563000	5.60015000	24.81880000

$[Li_2PF_6]^-$ (2710 fs)

Li	15.15800000	8.78756000	25.30370000
Li	12.87080000	8.38690000	25.43200000
P	9.77818000	7.03795000	25.64180000
F	14.13090000	9.16358000	26.66430000
F	11.06160000	8.41323000	25.08230000
F	10.99370000	6.46195000	26.57250000
F	9.18754000	8.14456000	26.66390000
F	14.17960000	8.01429000	24.09380000
F	8.75231000	5.83965000	26.38800000
